# Growth and Antifungal Resistance of the Pathogenic Yeast, *Candida Albicans*, in the Microgravity Environment of the International Space Station: An Aggregate of Multiple Flight Experiences

**DOI:** 10.3390/life11040283

**Published:** 2021-03-27

**Authors:** Sheila Nielsen, Kenna White, Kyle Preiss, Dakota Peart, Kathryn Gianoulias, Rachel Juel, James Sutton, James McKinney, Jaclyn Bender, Gabrielle Pinc, Kela Bergren, Wendy Gans, Jessica Kelley, Millard McQuaid

**Affiliations:** Department of Microbiology and Immunology, Montana State University, Bozeman, MT 59717, USA; Kenna.r.white@gmail.com (K.W.); k.preiss@plexusrt.com (K.P.); dakotap80@gmail.com (D.P.); kathryn.gianoulias@gmail.com (K.G.); racheljuel2014@gmail.com (R.J.); jamesbsutton@hotmail.com (J.S.); jcmckinney12@gmail.com (J.M.); jaclyn.jmp@gmail.com (J.B.); pincelle3@gmail.com (G.P.); bergren_09@hotmail.com (K.B.); wgans5401@gmail.com (W.G.); Jessbrown7@gmail.com (J.K.); mcquaid.millard@gmail.com (M.M.)

**Keywords:** microgravity, spaceflight, yeast, amphotericin B, caspofungin, antifungal, ISS, Space X, FPA, GAP, FEP bag

## Abstract

This report was designed to compare spaceflight-induced cellular and physiological adaptations of *Candida albicans* cultured in microgravity on the International Space Station across several payloads. *C. albicans* is a common opportunistic fungal pathogen responsible for a variety of superficial infections as well as systemic and more severe infections in humans. Cumulatively, the propensity of this organism to be widespread through the population, the ability to produce disease in immunocompromised individuals, and the tendency to respond to environmental stress with characteristics associated with increased virulence, require a better understanding of the yeast response to microgravity for spaceflight crew safety. As such, the responses of this yeast cultivated during several missions using two in-flight culture bioreactors were analyzed and compared herein. In general, *C. albicans* had a slightly shorter generation time and higher growth propensity in microgravity as compared to terrestrial controls. Rates of cell filamentation differed between bioreactors, but were low and not significantly different between flight and terrestrial controls. Viable cells were retrieved and cultured, resulting in a colony morphology that was similar between cells cultivated in flight and in terrestrial control conditions, and in contrast to that previously observed in a ground-based microgravity analog system. Of importance, yeast demonstrated an increased resistance when challenged during spaceflight with the antifungal agent, amphotericin B. Similar levels of resistance were not observed when challenged with the functionally disparate antifungal drug caspofungin. In aggregate, yeast cells cultivated in microgravity demonstrated a subset of characteristics associated with virulence. In addition, and beyond the value of the specific responses of *C. albicans* to microgravity, this report includes an analysis of biological reproducibility across flight opportunities, compares two spaceflight hardware systems, and includes a summary of general flight and payload timelines.

## 1. Introduction

*Candida albicans* (*C. albicans*) is a commensal fungus found throughout the human body in locations such as the mouth, gastrointestinal tract, vagina, and skin [1,2]. Most infections are superficial, such as fungal nails, yet as an opportunistic pathogen, this yeast is capable of causing severe, life-threatening illness in immunocompromised hosts; therefore, it is a potential concern for crew during long term spaceflight [3,4,5,6]. In addition, the dichotomy between the commensal and pathogenic behaviors of *C. albicans* is complex and the molecular events responsible for converting a normally benign commensal into a highly pathogenic organism, which in systemic infections can produce 30–45% mortality, have not been fully elucidated [7,8,9,10,11]. Moreover, the therapeutic repertoire to treat systemic *Candida* infections is limited [12]. There are four classes of antifungal agents used clinically, including polyenes (e.g., Amphotericin B), echinocandins (e.g., caspofungin), azoles (e.g., fluconazole) and nucleoside analogs (e.g., flucytosine) [13]. Amphotericin B (AmB) was utilized as an early therapeutic for *Candida* infections and its derivatives remain among the most effective treatments for systemic disease [14,15,16]. For these reasons, AmB was chosen for analyses in the microgravity-associated susceptibility studies described herein. It has been proposed that AmB functions at the level of the cell membrane, where it interacts with the yeast-specific sterol, ergosterol, forming pores and compromising membrane integrity [13,15]. In contrast, caspofungin (CAS) was used to evaluate the yeast response to an antifungal agent harboring a mechanism of action distinct from that of AmB. Caspofungin compromises cell wall formation by inhibiting (1–3)-β-D-glucan synthase [11]. 

*C. albicans* is a dimorphic eukaryotic organism consisting of spherical yeast form cells and elongated filamentous cells and has been shown to respond to environmental stresses such as temperature, pH, and altered gas environments with physiological characteristics often associated with virulence [17,18,19,20]. Indeed the extreme environment of microgravity presents a unique challenge for organisms that have evolved under normal gravity. Microgravity may provide novel environmental stress cues leading to physiological adaptations or may allow insights into molecular pathways otherwise masked by a gravity-based environment, such as that described for the Pseudomonas *Hfq* pathway [21,22]. Among the potential adaptations of interest are those properties of *C. albicans*, on both the single and multicellular level, that enable it to infect an immunocompromised host. A well-studied phenotypic transition in *C. albicans* is filamentation, or hyphal/pseudohyphal formation, which can be induced by a variety of environmental conditions including temperature, pH, serum, elevated CO_2_ and hypoxia [23,24,25]. The combination of filamentous and yeast form cells can also form biofilms, or multicellular communities, contributing to increased virulence and enhanced resistance to environmental insults. As such, *C. albicans*, particularly in community form, has been documented to have increased resistance to antifungal agents [4,26,27]. In short, this yeast is capable of altering its phenotype in a number of ways to become better suited for infection and the environmental cues responsible for initiating these transitions are numerous. Hallmarks of an infectious form of *C. albicans* include robust growth, filamentation, biofilm production and resistance to antifungal agents. The studies included herein will focus on these yeast characteristics to determine whether microgravity represents an environmental cue with the potential to alter virulence.

Due to limited access and the expense of spaceflight opportunities, ground-based simulators, such as rotating wall vessels or random positioning machines, have been used to generate a ‘modeled microgravity’ environment in order to predict the response of cells to spaceflight microgravity [28,29,30,31,32,33]. High Aspect Ratio Vessels (HARV) are rotating-wall vessels that, by revolving about an axis perpendicular to the gravitational vector, create a state of functional weightlessness and minimal fluid shear within the chamber. Low-fluid shear may indeed be an important environmental signal for organisms cultivated in microgravity. Reduced convection and diminished bulk flow may resulted in an altered cell-associated microenvironment through depletion of nutritional components and accumulation of waste products [34]. Interestingly, when cultivated in analog bioreactors or in the true microgravity environment, many, but not all, organisms display morphological and genetic changes consistent with increased virulence and invasive behavior [22,31,35,36,37,38]. Previous studies of *C. albicans* cultured in HARV bioreactors demonstrated adaptations that included cells with increased filamentation, increased resistance to the antifungal agent AmB, altered colony morphology, and altered biofilm complexity [29,31]. The studies described herein focus on whether exposure of *C. albicans* to true microgravity induces similar adaptation responses to those observed in ground-based analog studies, especially as they relate to the potential for increased virulence. Embedded in these studies are comparisons between spaceflight experiments and between two separate flight hardware systems.

## 2. Materials and Methods

### 2.1. Yeast Strain and Growth Conditions

*C. albicans* (SC5314, American Type Culture Collection) was maintained as a frozen stock at −80 °C. Routinely, and specifically the week before flight preparations, streak plates were initiated from frozen stock. As needed for experiment preparation, isolated colonies were used to initiate overnight planktonic cultures. For ground-based studies and flight sample preparation, all cells were cultured in YPD (1% yeast extract, 2% peptone, 2% glucose, Becton Dickinson, Franklin Lakes, NJ, USA) at 30 °C with shaking at 225 rpm for up to 18 h for planktonic cultures and inverted for 3 days for plated colonies, unless otherwise noted in specific methods. 

### 2.2. Missions: Common Procedures

**Figure 1:**
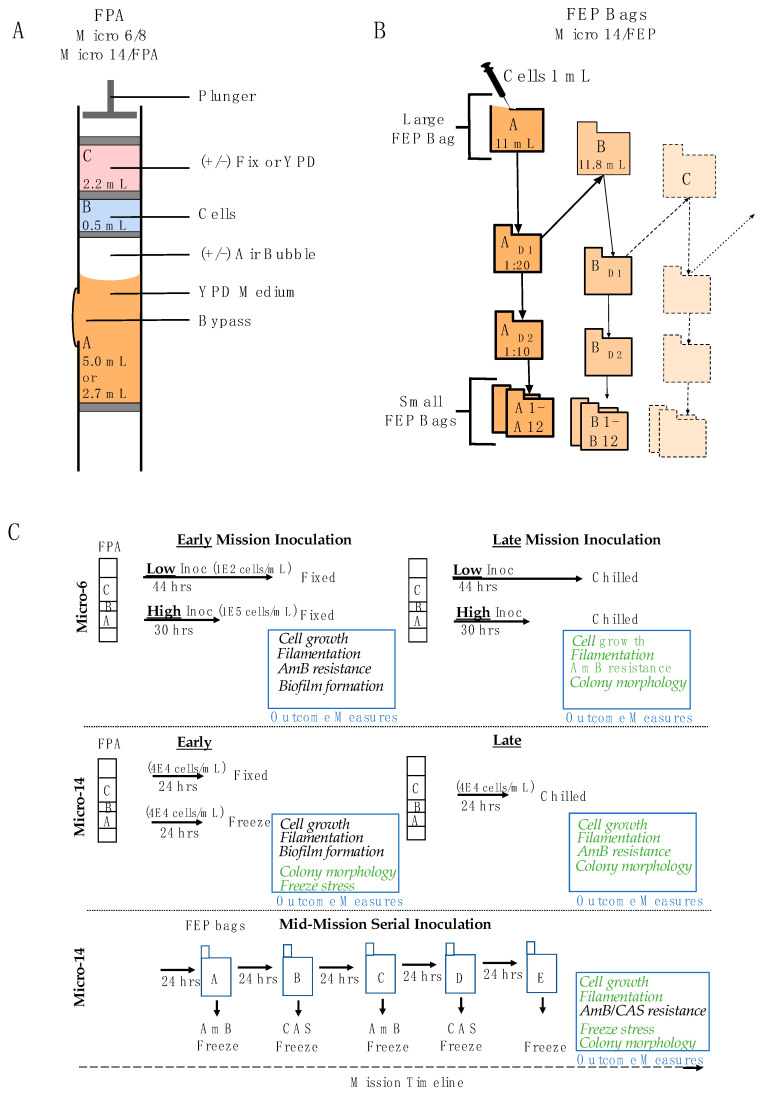
Schematic representation of the hardware and experimental designs used in the mission payloads. (**A**) FPA bioreactors were used for the Micro-6/8 and Micro-14/FPA payloads and were loaded with two or three chambers, loaded in sequence, and separated by rubber septa. Medium was loaded in chamber A (2.7 mL in three chamber design, 5 mL in two chamber design) and cell inoculum was loaded in chamber B (0.5 mL). In the three chamber design, chamber C was loaded with 2.2 mL of fixative or fresh medium. Chambers were sequentially mixed on a predetermined schedule by depressing a plunger, and consequently the column of reagents and septa, to allow mixing of reagents through the bypass in the glass barrel. All eight FPAs within a GAP were processed as a unit. At the conclusion of the experimental operations, the FPAs were stored within the GAP at the prescribed temperature. (**B**) FEP bags were used for the Micro-14/FEP payload, allowing for dilution (D_1_ and D_2_ bags) and serial subculture procedures. To activate growth, yeast (1 mL, 4 × 10^4^ cells/mL) was injected into the first Large FEP bag (Bag A containing 11 mL YPD) and cultured for 24 h +/− 1 h at 30 °C. At the end of this growth stage, an aliquot was removed and diluted 20-fold (Bag A_D1_). From this dilution, the next Large FEP bag was inoculated (0.25 mL into Bag B containing 11.8 mL YPD) and cultured for 24 h +/− 1 h at 30 °C. This process was repeated until the cells had been cultured for five days (through Bag E). On each of the first four days, samples were also prepared to analyze antifungal sensitivity. For these samples, the dilution series was extended from the D_1_ dilution bag to the D_2_ dilution bag (1:10) and aliquots (0.2 mL) from D_2_ were used to inoculate Small FEP bags (A1–A12, B1–B12, etc.) containing antifungal agents or control medium (2.8 mL). Each day following the 24 h growth period and any necessary sampling, FEP bags (Large FEP bags A–E, dilution D_1_ bags A–D, and Small FEP antifungal bags) were frozen at <−80 °C for the remainder of the mission. (**C**) An overview is provided to summarize the experimental details for each payload. Cell inoculation, time of cultivation, relative time within the mission, and storage conditions are provided. In addition, the outcome measurements employed for each payload are indicated. Outcome measures that were performed/completed during flight with cells fixed in microgravity are indicated in black text. Outcome measures that were performed using cells returned viable (chilled or frozen) and processed post flight in the home laboratory are indicated in green text.

Pre-flight sample preparation was done at Kennedy Space Center in the Space Station Processing Facility (SSPF). Freshly prepared yeast streak plates, and any specialized reagents or equipment, were shipped overnight to the SSPF laboratory. These reagents were shipped in replicate on consecutive days to minimize any complications of potential transport delays. The research team arrived approximately 10 days prior to launch, navigated the necessary badging and training requirements within the site-specific scheduling and availability, received the shipments of biologics, and began experimental preparations forthwith. Flight and terrestrial control samples, with a predetermined and condition-specific sample excess (spares), were prepared in bulk whenever possible. Following an incubation period, the bulk-prepared samples were inspection for contamination and other anomalies (bubbles, volume discrepancies, etc.), and removed from the population as necessary. From the remaining samples, flight and terrestrial control specimens were chosen and extra samples were deconstructed, cleaned, and sterilized should they be needed for reloading in the event of an extended launch delay. It should be noted that selection of flight and terrestrial control samples was essentially random other than verifying that flight hardware was imperfection free (no glass chips/cracks, no angled septa, etc.). Following launch, the terrestrial control samples were shipped overnight to the Montana-based laboratory at the appropriate temperature using phase change packaging as needed. Flight and terrestrial control experiments were conducted near synchronously; the offset time being determined before flight and allowing for communication of each procedure from the flight crew, to the Implementation Partner (BioServe Space Technologies, Boulder, CO, USA), and then to the research team. Mission-specific timing is included in Table 1. In addition, Figure 1A,B synopsize the hardware utilized in each mission as well as the general contents and construction of the experimental samples. Specific experimental design details will be provided in the payload-specific sections below and are summarized in Figure 1C. To clarify the payload nomenclature, flight experiments are provided with an organizational name, in our case by NASA Space Biology as part of their funded microbial series of studies (Micro-*x*), and the payloads become broadly referred to by these names. The payloads (experiments) are manifested on a spaceflight mission once preflight requirements have been met and flight resources (space on a launch vehicle, crew time, International Space Station (ISS)- based resources) become available. Our first set of experiments is referred to as Micro-6, which was flown on Space X Cargo Resupply Service (CRS)-1, with a reflight referred to as Micro-8, flown on Space X CRS-4. The next set of experiments (Micro-14) was split between two missions so is further defined, for the benefit of clarity herein, by the hardware used, specifically the Fluid Processing Apparatus (FPA) and Fluorinated Ethylene Propylene (FEP) bags. Micro-14/FPA was flown on Space X CRS-16 and Micro-14/FEP was flown on Space X CRS-17. The details of each payload/experiment are described in detail below. In each case, the *C. albicans* inoculum was prepared and stored in sterile water to maintain stasis. Experiments were initiated, or activated, by the addition of the inoculum to medium to stimulate growth. Following the defined growth period, experiments were terminated by the addition of fixative or by placing the samples at a restricted temperature (−95 °C or 4 °C, depending on the experiment). Upon completion of the mission and once the samples were received in the home laboratory, yeast were isolated from the hardware, which in many cases required deconstruction of the hardware components (de-integration), and processed as indicated for each of the specific outcome measures. 

### 2.3. Mission-Specific Growth Conditions and Procedures

#### 2.3.1. Micro-6 (and the Reflight Micro-8), Space X CRS-1 and -4, Respectively

**Figure 2:**
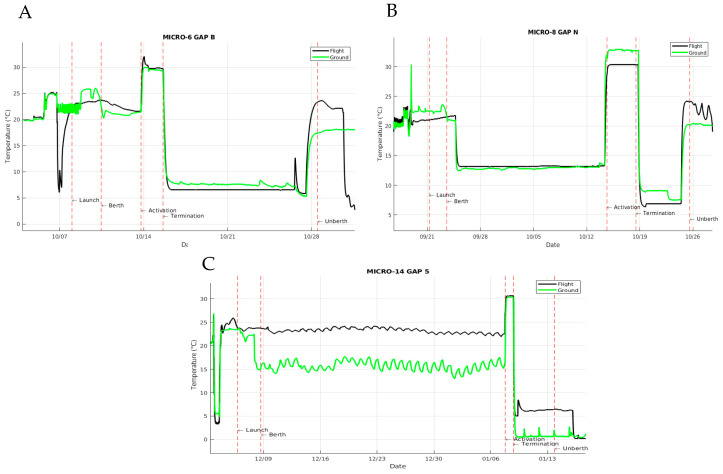
Temperature tracings comparing the experience of flight and terrestrial control samples throughout the duration of the missions. HOBO temperature sensors were incorporated into selected GAPs and retrieved during hardware de-integration at the end of the mission. (**A**) Samples in the Micro-6 payload that were activated early in the SpX CRS-1 mission, (**B**) Samples in the Micro-8 payload that were activated and then terminated late in the SpX CRS-4 mission, and (**C**) Samples in the Micro-14/FPA payload that were activated and ‘terminated’ with fresh medium late in the SpX CRS-16 mission for return of viable cells. Temperature sensors were not associated with FEP bag bioreactors in SpX CRS-17. Terrestrial control operations were performed near synchronously (2 h delay from flight) and for the purpose of this illustration the time offset was removed from the tracings to allow overlap of transitions for better comparison. Flight temperatures are traced in black, terrestrial control temperatures are traced in green, and key operations are indicated in text and highlighted with red vertical lines.

The hardware utilized for these payloads consisted of FPAs, organized eight per Group Activation Pack (GAP) (BioServe Space Technologies). See Figure 1A for a graphic representation of the FPA hardware and Figure 1C for an overview of the experimental design. Each FPA was divided into 2 or 3 chambers, indicated as chamber A, B and C, which were loaded sequentially and compartmentalized by rubber septa. Chamber A was loaded with 2.7 mL 1.2× YPD (Sigma Aldrich, St. Louis, MO, USA), containing the antifungal agent AmB (Sigma Aldrich, St. Louis, MO, USA) where indicated. Chamber B was loaded with 0.5 mL *C. albicans* diluted in water (Low inoculum = 100 cells/mL, High inoculum = 1 × 10^5^ cells/mL, and are referred to as Low and High, respectively). Chamber C contained 2.7 mL termination medium, consisting of either fixative (8.4% *v*/*v* paraformaldehyde, Polysciences, Inc, Warrington, PA, USA) or fresh medium (1x YPD for samples returned live). The GAPs were maintained at a cool temperature, specifically 4 °C pre-flight, ambient during launch, and 12 °C on the ISS, until the scheduled activation at 30 °C. Following the growth period (44 h for Low inoculum and 30 h for High inoculum), GAPs were chilled to 4 °C and maintained at that temperature through the remainder of the mission. There were two operational windows during the mission. Samples that were scheduled to be fixed were activated early in the mission (referred to as Early) to avoid any potential decreases in viability due to on-orbit storage. Conversely, cells that were scheduled to be returned live were activated as late as possible in the mission (referred to as Late) so as to retain viability following the growth period and during the transition back to Earth and the home laboratory. During transport back to Earth (splashdown in the Pacific Ocean) and then to the California-based handover location, samples were at ambient temperature (Micro-6) or 4 °C (Micro-8). Following payload handover to the Implementation Partner (BioServe Technologies) and research team, the samples were transitioned to storage on ice until they arrived at the home laboratory. Viable samples were isolated from the de-integrated hardware and processed the day of arrival. HOBO temperature sensors were included in representative GAPs to retrospectively analyze the temperature profiles and consistency between flight and terrestrial control samples (Figure 2).

#### 2.3.2. Micro-14/FPA, Space X CRS-16

Similar to Micro-6/8, the hardware utilized for this payload consisted of FPAs organized eight per GAP (BioServe Space Technologies). This experiment also had two operational windows. Some samples were cultured as described below early in flight (Early) and preserved either with the addition of paraformaldehyde or by freezing until the end of the mission. The remaining samples were maintained at 4 °C and cultured as late as possible in the mission (Late) and returned live and chilled (4 °C) for post flight analyses requiring cell growth. During each operational window, cells were cultured in microgravity for 24 h +/− 1 h at 30 °C. FPAs were prepared with 2 or 3 chambers as follows. (1) Samples to be frozen consisted of 2 chambers: Chamber A was loaded with 5 mL of YPD (referred to as Low due to the lower cell concentration upon inoculation) and overlaid with an air bubble to promote gas exchange. Chamber B was loaded with 0.5 mL of yeast suspended in water (4 × 10^4^ cells/mL). There was no chamber C. (2) Samples to be fixed consisted of 3 chambers: Chamber A was loaded with 2.7 mL YPD (referred to as High as the cell concentration was higher at the time of inoculation) and overlaid with an air bubble. Chamber B was loaded with 0.5 mL of yeast suspended in water (4 × 10^4^ cells/mL). Chamber C was loaded with 2.2 mL paraformaldehyde (10% *v*/*v*). (3) Samples to be returned viable consisted of 3 chambers: Chamber A was loaded with 2.7 mL YPD (referred to as High as the cell concentration was higher at the time of inoculation) and overlaid with an air bubble. Chamber B was loaded with 0.5 mL of yeast suspended in water (4 × 10^4^ cells/mL). Chamber C was loaded with 2.2 mL 1× YPD. Following completion of the mission, the live samples were isolated from de-integrated hardware and processed the day they arrived in the home laboratory; fixed and frozen samples were isolated shortly thereafter. Temperature sensors were included in representative GAPs to retrospectively analyze the operational transitions as well as consistency between flight and terrestrial control samples (Figure 2).

#### 2.3.3. Micro-14/FEP, Space X CRS-17

The FEP bag hardware was utilized for cultivating yeast in this payload and involved serial passage-style growth. The parent culture (Large FEP) was serially propagated over five days and from each of these populations a set of antifungal sensitivity cultures were initiated (Small FEP, see Figure 1B and details in Section 2.4.3. Large FEP bags for the parent cultures were filled with 1× YPD (11.0 mL for the initial culture, Bag A, and 11.8 mL for the four downstream cultures, Bags B-E) and gas bubbles were removed. The yeast inoculum of 1 mL (4 × 10^4^ cells/mL in water) was loaded into a 3 cc syringe and sealed for transport. The needleless syringe was used to inoculate the initial culture (11 mL FEP bag, Bag A) on orbit. Following 24 h of growth at 30 °C, 0.5 mL of culture was removed from Bag A and diluted 20-fold in water (D_1_ FEP bags). An aliquot of this diluted sample (0.25 mL) was used to inoculate the next Large FEP bag (11.8 mL YPD, Bag B). This process was repeated such that the yeast was continually propagated through five cycles of 24 h growth (Bags C, D and E). The Large FEP bag samples were frozen (<−80 °C) either in the presence (Bag A and E) or absence (Bags B–D) of glycerol as a cryopreservation agent. As a complement to this series of growth, Small FEP bags containing 2.8 mL YPD and antifungal agents as indicated (AmB, 0.12–5 mg/L; and CAS, 0.01–0.1 mg/L) were inoculated with a dilution of the corresponding Large FEP culture. Specifically, the 20-fold dilution prepared from the large FEP bag as described above (D_1_) was further diluted 10-fold (D_2_ FEP bag) and aliquots (0.2 mL) were used to inoculate each Small FEP bag (A_1–12_ from FEP Bag A; B_1–12_ from FEP Bag B, etc.). These cultures were grown at 30 °C for 24 h and then frozen at <−80 °C for the duration of the mission. At the end of the mission, frozen samples were maintained at <−25 °C from undocking to the California-based handover location using phase change bags (Cold Stowage, NASA, Houston, TX, USA) and then to the home laboratory using dry ice. 

### 2.4. Ground-Based Analyses

#### 2.4.1. Cell Growth and Morphology

Cells were fixed with paraformaldehyde (4% *v*/*v*, final) either in flight or immediately following hardware de-integration post flight. Direct manual cell counts were performed in duplicate using a Bright-Line hemocytometer (Sigma Aldrich) quantifying total cells as well as the contribution of cells with an extended morphology (filamentation; length greater than 2× the width of the cell) to the population. Samples were also stained with Calcofluor White (25 μM final, Molecular Probes) to stain chitin in the yeast cell wall and visualized using epifluorescence microscopy. Cell diameter and filament length were recorded using image collection on a Nikon Eclipse E-800 and interpreted using the software program Metamorph.

Samples of non-fixed cultures were cultured post-flight on YPD agar plates as an indication of viability. Viability was calculated using colony forming units (CFU) with the actual/observed number of colonies compared to that expected based on cell counts. It is important to note that cell viability is likely to be higher than interpreted from the CFU analyses included in these studies due to the convention of our cell counting; budding cells are counted as two cells since that is how they will likely contribute to growth in planktonic cultures, whereas, these cells may remain associated during the plating process and give rise to a single colony. With this in mind, it should be understood that post-flight cell viability was quite high.

#### 2.4.2. Biofilm formation

Previous studies demonstrated that *C. albicans* formed biofilm communities on the siliconized rubber membrane component of HARV bioreactors [31,39]. For this reason, small (5 mm^2^) samples of this membrane (Synthecon, Houston, TX, USA) were included in chamber A of select FPAs in Micro-6 and Micro-14/FPA. Samples were fixed during the mission and analyzed upon return. Upon hardware de-integration, the membrane coupons were placed into individual wells of a 24-well plate and stained with Crystal Violet (0.4% *w*/*v* in water, 1 mL per well, Sigma Aldrich) to stain cells and cell matrix components, adapted from previous methods [40]. Excess stain was aspirated, 1mL water was added to each well and mixed thoroughly. From each well, 100 μL was transferred to a 96-well plate to record optical density (referred to as ‘stain’). Excess water was removed from each well and discarded. A fresh aliquot of sterile water (1 mL) was added to each well, mixed, and a 100 μL sample was transferred to the 96-well plate (wash 1). Membranes were subjected to two more rounds of water washes and a final extraction with 1 mL of 10% *v*/*v* acetic acid (aa wash) to remove residual organic material, each involving transfer of a 100 μL aliquot to the 96-well plate (referred to as wash 2, wash 3, and aa wash, respectively). Stain levels were quantified on a microplate reader at 595 nm (iMark, BioRad, Hercules, CA, USA). 

#### 2.4.3. Antimicrobial Resistance 

The susceptibility of *C. albicans* to antifungal agents was determined by the ability of cells to grow in the presence of the indicated drug. Cells were either cultured in the presence of antifungal agents during the mission and preserved by fixation or freezing, or following the flight, live yeast was exposed to the antifungal agents immediately after recovery from the hardware. In the former case, the growth medium was supplemented with the antifungal agent in the hardware and maintained at 4 °C to retain drug efficacy into the mission timeline. Following the 24 h growth period, samples were fixed as indicated in the Mission-Specific details. Susceptibility was determined by direct cell count and the results were compared to samples without drug, as well as to the corresponding terrestrial controls. Alternatively, yeast cells were cultured in microgravity without treatment and returned live. Viable cells were recovered from the de-integrated hardware, counted, diluted to 1 × 10^5^ cells/mL, and added (100 μL) in triplicate to wells of a 96-well plate pre-loaded with 100 μL control medium (2× YPD) or antifungal agent (2×) in YPD. The plates were incubated at 30 °C for 24 h and 72 h and cell growth was quantified by optical density at 595 nm (iMark, BioRad, Hercules, CA, USA). The drug concentrations chosen to assess AmB and CAS sensitivity were identified in the literature and refined empirically based on dose sensitivity assays performed with the Candida isolate used in the laboratory (unpublished data and [31,41,42,43,44]).

#### 2.4.4. Statistical Analysis

For all data sets, means +/− standard deviations are shown. Determination of the difference between mean values for each data set was assessed by Student *t*-test. Significance testing comparisons are described in the associated figure legends and are routinely provided as flight compared to terrestrial controls, antifungal agent to control, or between hardware types. Significance was established at *p* values <0.05, with more specific *p* values provided in the corresponding figure legends. 

## 3. Results

### 3.1. Mission Timelines

Although each mission has different timing, the specifics of which change in real time based on the actual launch date and accompanying mission scenario, Table 1 summarizes our general experiences for each payload. Categories in this table were chosen to provide perspective and demonstrate the temporal opportunities and constraints in which experiments are conducted. Understanding the launch cancelation (scrub) and subsequent launch scenarios (*Scrub delay* and *Launch date*) establishes the prelaunch experiment planning and preparation, especially as they relate to sensitive reagents and cells that have longevity and storage considerations. *Launch to dock* (arrival at the ISS) and *Dock to sample access* represent times during which samples are largely inaccessible for processing, although temperature requirements can often be maintained even with non-powered payloads. The lapse of time between when the vehicle docks and when the crew accesses specific samples may be important for an experiment, but occurs during a busy time of the mission. As such the timing for sample transfer to on-orbit power-controlled environments or early initiation of experimental procedures are negotiable, but ultimately needs to fit within the crew schedule, competing requests, and mission priorities. The *Length of mission* illustrates the active duration of the on-orbit procedures during which experiments can be conducted, including the shoulder days necessary for vehicle unpacking (beginning of the mission) and packing (end of the mission), during which crew time may be limited. Of note, samples often need to be equilibrated to a particular storage/post-mission temperature for travel once the experimental procedures are complete. One-to-two days is typically allowed for this temperature transition before packing the samples into the return vehicle (*Experiment completion to undock*). Finally, from the time the vehicle splashes down to when the samples arrive at the home laboratory, *Splashdown to home lab*, is relevant for sample processing considerations. These timelines will vary based on the splashdown location, travel time to shore, requirements of the science, and individual opportunities. In total, the timeline data is presented to provide some perspective as to how to prepare for and maximize the success of a spaceflight experiment using the ISS.

### 3.2. Sample Temperature Transitions throughout Mission

Our first flight experiment was transported to the ISS on SpX CRS-1, so we were eager to obtain as much tracking information as possible, especially since there was no historical data on which to depend. In order to evaluate whether samples were exposed to the expected temperatures and to compare ambient temperatures within the ISS to those of the terrestrial setting of our laboratory, HOBO temperature sensors were included in selected GAPs. Figure 2 provides representative temperature tracings for flight and corresponding terrestrial controls for the duration of the indicated mission. Examples from SpX CRS-1 (Figure 2A), -4 (Figure 2B) and -16 (Figure 2C) are included. SpX CRS-17 utilized FEP bags and thus did not accommodate an accompanying temperature sensor. As illustrated in each panel, the active aspects of the experimental procedures were well aligned between the flight (black lines) and terrestrial controls (green lines) in terms of time and temperature (see activation and termination indicators on tracings, labeled and indicated by red vertical lines). The time offset (usually 2 h) between flight and terrestrial experimental procedures, which is necessary to verify on-orbit procedures by the crew, was removed to improve the temperature comparisons between conditions. Although the mid-mission storage temperatures varied slightly, with terrestrial controls elevated 1–2 °C relative to flight in SpX CRS-1 (Figure 2A) and reduced by approximately 7 °C in SpX CRS-16 (Figure 2C), the sample quality was likely to be minimally affected because the temperature variance was during stasis or after the operational window of the experiment. Of concern though, was the elevated temperatures of the SpX CRS-1 flight samples following undocking (approaching 25 °C) since the elevated temperature would promote post-microgravity growth of samples being returned live. Other than that specific situation, the temperatures of terrestrial samples were well-aligned with flight samples and the temperature targets were well approximated.

### 3.3. Yeast Growth

**Figure 3:**
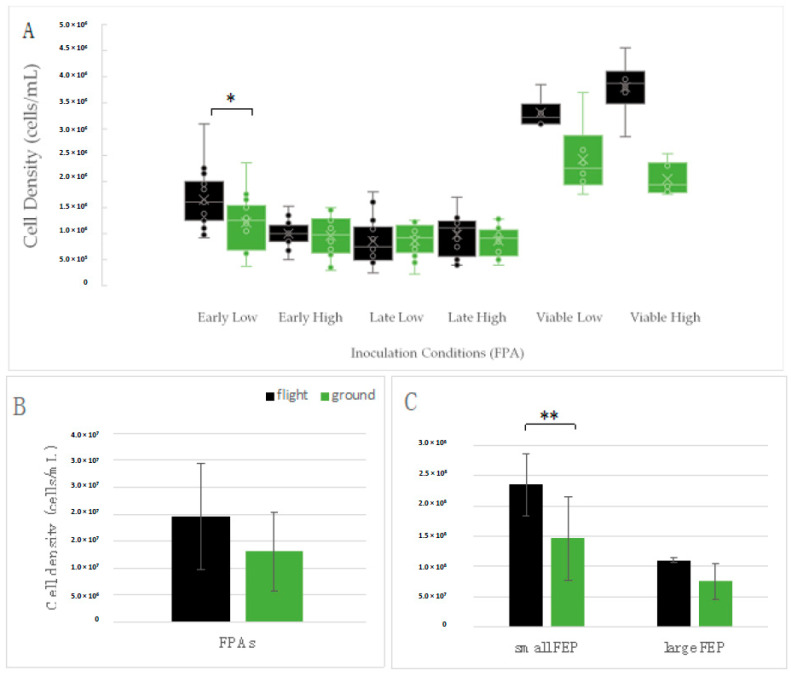
Cell growth determination using direct cell counts at the end of each of the three missions. Cell growth was evaluated in the (**A**) Micro-6 (FPA), (**B**) Micro-14/FPA, and (**C**) Micro-14/FEP payloads. These analyses allowed mission-to-mission comparison using the same hardware (FPA comparison between **A**,**B**), as well as comparison using two different types of spaceflight hardware (**A**/**B** = FPAs versus **C** = FEP bags). ‘Low’ refers to the samples seeded with a low inoculum, ‘High’ refers to samples seeded with a high inoculum, ‘Early’ refers to samples activated near the beginning of the spaceflight mission, ‘Late’ refers to samples activated late in the spaceflight mission, and ‘viable’ refers to samples returned alive. Differential growth was not noted in the viable samples since flight cells were subjected to different transport temperatures than the terrestrial controls. Large FEP bags represent those with a final volume of 12 mL; small FEP bags represent those with a final volume of 3 mL. Black bars = flight; green bars = terrestrial controls. * *p* = 0.02; ** *p* = 0.01.

At the time the initial studies were designed and initiated in 2011, one priority was to determine whether microgravity influenced overall cell growth. Although a simple outcome measure, growth was an important consideration for yeast as a potential infectious risk in spaceflight, but also provided insight into yeast adaptation to the microgravity environment. Embedded within the growth studies was a plan to allow the yeast to grow in microgravity for as long as possible, in spite of the requirement for a sealed growth chamber to ensure crew safety in the presence of an opportunistic pathogen. As such, the FPA bioreactors were prepared with two concentrations of yeast; a high inoculum (High, 1 × 10^5^ cells/mL) to ensure growth, and a low inoculum (Low, 100 cells/mL) to allow maximum replicative potential before stationary phase was reached. The protocol was also designed such that yeast growth was activated early (Early) in the mission to minimize any potential storage stress on the cells while maintained in water-induced stasis. In addition, comparable samples were stored and activated late (Late) in the mission so that cells could also be returned live for post-mission analyses. This design also allowed some comparison between cells activated Early and Late in the mission to evaluate the effects of storage in the microgravity environment. As seen in Figure 3A, cell growth in most of the samples peaked at ~1 × 10^6^ cells/mL, although the Low inoculum samples activated Early in the mission (Early Low) grew to a slightly higher density and demonstrated a minor advantage to cells grown in microgravity (Early Low, * *p* = 0.02).

It is worth noting that in Micro-6, cells that were scheduled to be returned live and stored at 4 °C actually experienced a 3-day transport temperature of nearly 25 °C (Figure 2). The cell growth data in Figure 3A for viable samples (‘Viable Low’ and ‘Viable High’) suggest that the cells indeed continued to grow. The higher level of growth in the flight samples as compared to terrestrial controls likely reflects the elevated temperature during transport. There was not a mechanism to track temperatures in real-time in order to subject terrestrial controls samples to conditions comparable to that experienced by the flight samples. Interestingly, the additional growth of the viable samples in both microgravity and terrestrial conditions indicates that the culture environment was capable of supporting at least 1–2 additional doublings, thus other factors must have been contributing to the apparent growth cap (1 × 10^6^ cells/mL) observed in the other Micro-6 samples (Figure 3A). For clarity, the on-orbit growth period was sufficiently long for the Low inoculum samples to reach a density of 6 × 10^7^ cells/mL and the High inoculum samples to reach a density of >1 × 10^8^ cells/mL, using generation times established in ground-based studies.

As seen in Table 2, generation times were calculated, factoring in the differences in inoculum size, and found to be similar between flight samples and the terrestrial controls. Interestingly, samples with the High cell inoculum had much longer generation times (300+ min/generation as compared to 150+ min/generation), suggesting the cell density of 1 × 10^6^ cells/mL was reached and the culture likely went into stationary phase. A kinetic analysis of cell growth was not performed due to resource constraints and safety precautions, so temporal aspects of the yeast growth dynamics cannot be concluded from these studies. 

In the Micro-14 experiments, yeast grew to a 10-fold higher cell density than in the Micro-6 payload, even though they both used the FPA hardware (Table 2, Figure 3B). As indicated in Table 2, the generation times were similar among the Micro-14/FPAs, regardless of the initial cell concentration. Due to the similarity in growth characteristics of the Low and High inoculum samples in the Micro-14/FPA payload, the data were aggregated for Figure 3B. In this case, there is a trend toward an increased cell density of cells cultivated in microgravity. 

Finally, yeast cells grew *most robustly* when cultured in the FEP bag bioreactor (Table 2, Figure 3C) both in terms of shortest generation times and highest cell density. Indeed, the cell density observed in the FEP bags resembles that obtained with ground-based shaker cultures (2–3 × 10^8^ cells/mL). For comparison, activation occurred at approximately 4100 cells/mL in the Large FEP bags (parent cultures for the serial cultivation) and approximately 33,300 cells/mL in the Small FEP bags (medium-only control bags from the antifungal drug study). These inoculations were the result of serial dilutions and inoculation done by flight crew (and corresponding ground crew), so cell concentrations are predicted. The cells within the Large FEP bags likely did not reach maximal density due to the length of the growth period, which in hindsight could have been extended another couple of hours. Data obtained from the small FEP bags in this payload indicates that *C. albicans* grows to a higher density in microgravity than in the terrestrial controls (** *p* = 0.01). Cumulatively, there was a trend in each of the payloads and in two types of hardware suggesting yeast grows to a higher cell density in microgravity than in terrestrial control conditions. 

### 3.4. Biofilm Formation

**Figure 4:**
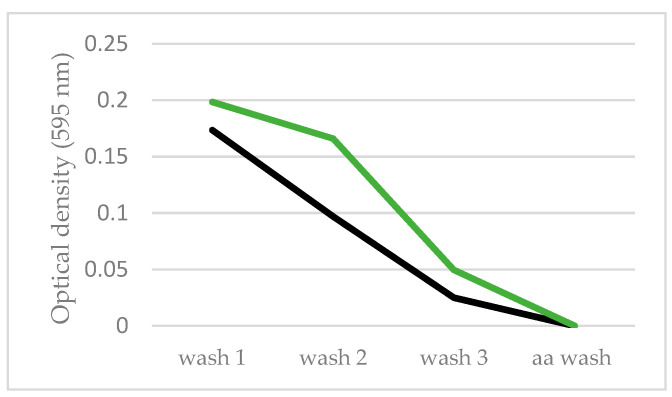
Passive seeding of biofilm communities on siliconized rubber membrane coupons. Siliconized rubber membrane coupons were included in the medium (chamber A) of selected FPAs in Micro-14/FPA and allowed to freely seed during the 24 h growth period of the experiment. Samples were fixed in microgravity and following sample return and hardware de-integration, the membranes were recovered and stained with Crystal Violet as an indicator of cells, cellular debris, and extracellular matrix. Stain that is retained on the membrane through a series of three water washing steps (wash 1, 2 and 3) and a final acetic acid wash (aa wash) is suggestive of a tighter integration of the cells with the substrate material. The stain removed by each wash was quantified by optical density at 595 nm. Black line = flight; green line = terrestrial control.

Biofilm formation is an important adaptation for cells facing environmental stress and our previous studies indicated that biofilm communities formed on the siliconized rubber membranes in HARV bioreactors [31]. As such, small coupons of this material were included in select FPAs in the Micro-6 and Micro-14 payloads to determine whether C. albicans would colonize the substrate de novo during short duration growth. During post-mission analyses, colonies could not be identified by microscopy in either experiment. The membrane coupons included in Micro-14/FPAs were fixed during flight and upon return they were subjected to a staining and washing procedure that is designed to identify cells, cellular debris, and/or cell-deposited matrix attached to the membrane substrate. From published studies, the more resistant the stain is to removal by washing, the tighter the association is between the stained material (cell community and components thereof) and the substrate [40]. Although a small and preliminary experiment, Figure 4 indicates that there does not seem to be an advantage for cells to form biofilms on the membrane coupons under the conditions in which they were grown. It is important to note that these were 24 h growth studies and it may take longer for the yeast to form cellular communities, especially if the substrate is not specifically inoculated. Indeed, the terrestrial control membranes appeared to be more apt to retain stained cellular components than in the flight samples, perhaps due to gravity-induced sedimentation (green line). In contrast, yeast was retained to a greater extent on the membrane coupons of cultures grown in hypoxic conditions in microgravity than comparable terrestrial controls (manuscript in preparation). Longer term studies, with cells under a variety of conditions, will be required to fully understand biofilm formation in the microgravity environment.

### 3.5. Cell Morphology and Filamentation

**Figure 5:**
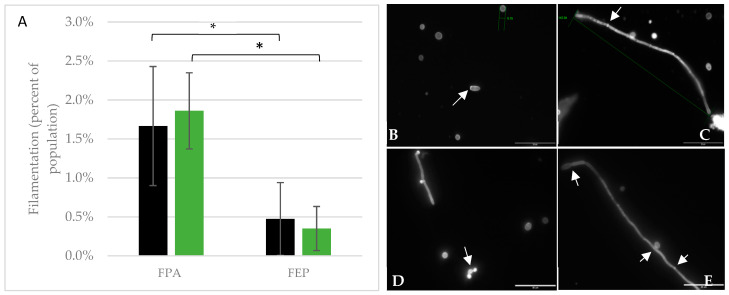
Quantification of yeast filamentous cell morphology. Aliquots from each growth condition in the three payloads were fixed and retained at 4 °C for microscopic analyses. Some samples were fixed in microgravity, but for those samples returned frozen or viable, the aliquot was fixed immediately upon sample return to the home laboratory. (**A**) Filaments were quantified as a percentage of the total population. In this analysis, samples were aggregated by hardware, thus the FPA data represents samples cultured in YPD from the Micro-6 and Micro-14/FPA payloads. Similarly, the FEP data represents the aggregate from samples cultured in YPD in Large FEP bags and Small FEP bags from the Micro-14/FEP payload. * *p* < 0.003 for FPA vs. FEP. (**B**–**E**) Calcofluor White stained cells were visualized to evaluate cell morphology from Micro-14/FPA, scale bar = 35 μm; (**B**,**C**) are from terrestrial control samples and (**D**,**E**) are from flight samples. The arrows highlight key features in each of the images, including, (**B**) a cell with a bud scar, as indicated by a cell surface region with higher intensity staining and a second cell with a caliper measurement of 5.1 μm, (**C**) a caliper measured filament (142 μm) in which higher intensity staining also indicates intercellular septa (arrow), (**D**) cells in the process of budding (arrow), and (**E**) long filamentous cell demarcated with narrowed septal junctions (top arrow), a cell budding from the filament (middle arrow), and increased staining at septal junctions (bottom arrow).

The propensity of *C. albicans* to form filaments when confronted with environmental stresses, and the association of this morphology with increased virulence, led us to evaluate cell size and shape as first line analyses to determine whether and how the yeast adapt to a novel environment. Indeed, we and others have demonstrated differences in cell size, cell clumping, and filamentation of *C. albicans* cultivated in simulated or true microgravity [29,35,36,45]. Initial analyses of the Micro-6 payload suggested that more of the samples cultured in microgravity contained filamented cells than did the corresponding terrestrial control samples. A more thorough analysis of each payload found substantial variability, but did not support the hypothesis that microgravity stimulates a filamentous morphology (Figure 5A). Interestingly, although the overall level of filamented cells was low in each of the payloads, cells cultured in FEP bags had a significantly lower level of filamented cells than those in FPAs, regardless of whether they were cultured in microgravity or on Earth (Figure 5A). Cell size was also variable, with large cells measuring over 7.5 μm and smaller cells measuring 3.1 μm, although the average size of yeast form cells was not significantly different between flight (4.8 μm) and terrestrial controls (4.6 μm). Filaments were observed within the populations, whether cultivated in microgravity or terrestrially, with some filaments reaching lengths of over 142 μm (Figure 5C). In contrast to previous studies [28,35], cell clumping was not observed.

### 3.6. Characteristics of C. albicans Cultured Post Flight

Many outcome measures can be obtained from experiments conducted to completion on the ISS, yet for reasons of feasibility (especially working with a potential pathogen), as well as sample access and efficient use of crew time, we were interested in determining whether samples could be cultured in microgravity, returned live, and assessed immediately upon return with ground-based procedures. As preliminary evidence from the data obtained on SpX CRS-1 and presented in Figure 3A, it was clear that yeast transitioned to 4 °C following the growth period in microgravity remained viable and were capable of continued growth. In those studies, viable samples from the Micro-6 payload were scheduled to be returned at 4 °C, but the unavailability of cold storage resulted in transition to ambient temperature, as illustrated in Figure 2A. The yeast continued to grow during transport as observed in Figure 3A (‘Viable Low’ and ‘Viable High’). In retrospect, it was determined that flight samples were maintained at approximately 5 °C higher than terrestrial controls for nearly three days, likely contributing to the relative higher growth observed in the flight samples. 

**Figure 6:**
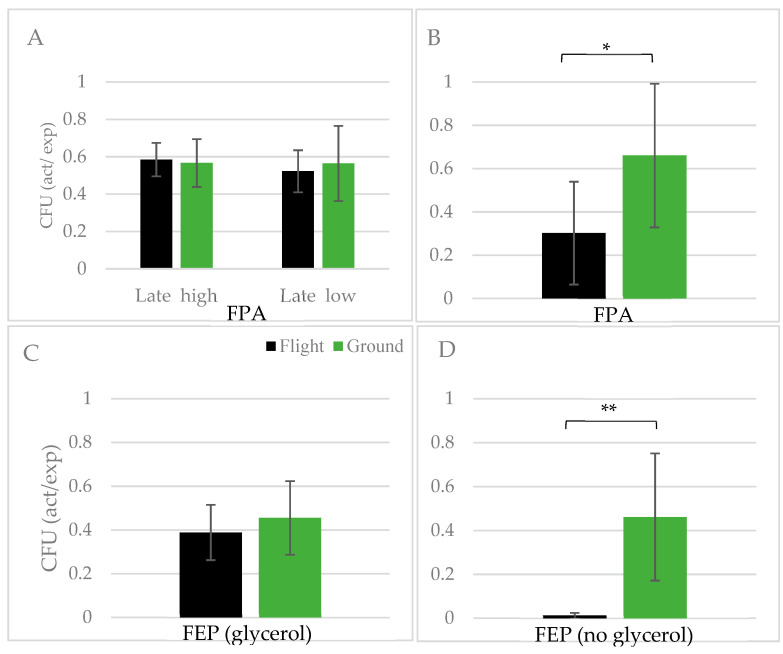
Viability of yeast cells stored chilled or frozen. At the conclusion of the growth period during the mission, cells were chilled to 4 °C (**A**) Micro-6 and (**B**) Micro-14/FPA. Alternatively, cells were frozen at <−80 °C and transported to Earth at <−25 °C with (**C**) glycerol as a cryopreservation agent in Micro-14/FEP, Large FEP bags or (**D**) without glycerol in Micro-14/FEP, Small FEP bags. Following sample return and hardware de-integration, cells were immediately isolated, counted, diluted, and plated to quantify colony formation. Colonies were counted after 3 days of growth at 30 °C. Data is represented as the actual number of colonies relative to that expected based on cell counts. Panel A represents samples activated late in the mission (Late) with either the high inoculum (High) or low inoculum (Low), as previously described. Black bars = flight; green bars = terrestrial controls. * *p* < 0.03, ** *p* = 0.003, flight vs. terrestrial.

As a quantitative analysis of post-flight viability, yeast cells were plated to evaluate colony forming units (CFU) relative to that expected based on direct cell counts. As illustrated in Figure 6A, the colony forming potential, and thus cell viability, following the Micro-6 mission was similar between flight and terrestrial controls, regardless of inoculum concentration. Interestingly, when the same analysis was performed on samples in the Micro-14/FPA payload, with transport temperatures maintained at <6 °C, terrestrial controls had a rate of viability double that of samples cultured in microgravity (Figure 6B). CFU analysis was also performed on yeast that were cryopreserved (5% glycerol) during the Micro-14/FEP mission. As observed in Figure 6C, the viability of cryopreserved yeast following propagation in microgravity is comparable to that of terrestrial controls. Although cryopreservation is a well-documented mechanism to maintain viability, pre-flight ground-based studies indicated that *C. albicans* is surprisingly hardy when frozen in the absence of a cryopreservation agent. Non-flight associated, ground-based studies demonstrated that although there is a rapid reduction in viability of cells stored at −80 °C, 50% of cells survive when frozen for 50 days, and 30% survive frozen for 100 days when compared to cells stored for the same amount of time in 5% glycerol (unpublished data). Considering this resiliency, cells frozen in microgravity without cryopreservation in Micro-14/FEP (Small FEP bags) were plated to determine viability. Surprisingly, yeast cultured in microgravity were remarkably more susceptible to freeze damage in the absence of a cryopreservation agent than were the terrestrial controls (Figure 6D). Similarly, flight samples frozen without cryopreservation in Micro-14/FPA also had a 5.9- to 10-fold lower viability (as per CFU) than did the terrestrial samples (data not shown). The discrepancy of survival between flight and control samples observed in these studies is intriguing. Due to reduced convection, the samples in microgravity are likely to freeze at a slower rate than terrestrial controls, yet a slower rate of freezing is often associated with improved cell viability [46,47]. Thus, the freezing rate is not likely to explain the difference in survival between microgravity and terrestrial cells. Indeed, ground-based studies comparing fast and slow freezing rates did not result in differences in yeast viability (data not shown). In addition, since the cells in Figure 6D were frozen for the return trip to Earth, it seems unlikely that re-entry *g*-force would have much impact on cell viability. Cumulatively, the diminished post-flight survival of cells cultivated in microgravity and stored either chilled (Figure 6B) or frozen without cryopreservation (Figure 6D) may suggest cellular adaptations that compromise cell wall or plasma membrane integrity. Current studies are underway evaluating changes in ergosterol, the membrane-associated yeast lipid comparable to cholesterol, levels in microgravity.

**Figure 7:**
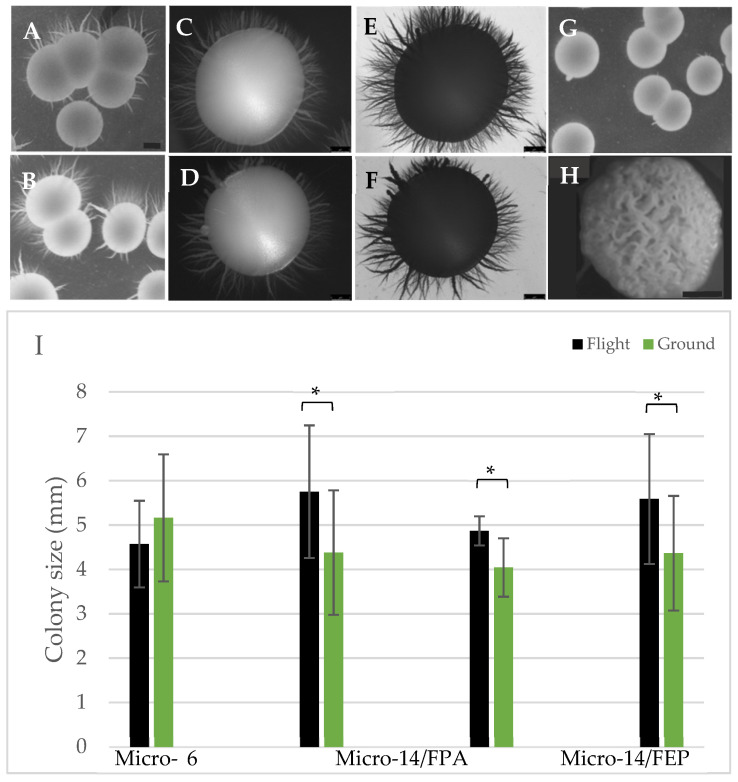
Colony morphology and size evaluated in yeast samples returned live. Following each mission, cells were isolated immediately following hardware de-integration, counted, diluted, and spread on YPD agar plates. Following 3 days of growth at 30 °C the plates were transferred to 4 °C pending analysis. (**A**–**H**) Individual colonies were imaged to evaluate colony architecture. (**A**,**C**,**E**,**G**) represent flight samples; (**B**,**D**,**F**) represent terrestrial controls; **H** represents the HIW colonies produced after cultivation in the ground-based HARV bioreactor. Specifically, colonies are associated with the following payloads (**A**,**B**) Micro-6; (**C**,**D**) Micro 14/FPA, view of top of colony; (**E**,**F**) Micro 14/FPA, view of bottom of colony; (**G**) Micro-14/FEP and (**H**) HIW colony from HARV bioreactor as a morphology reference. Scale bars in panels (**A**,**B**) = 2.25 mm, (**C**–**F**) = 1 mm, (**G**,**H**) = 2 mm. (**I**) The diameter was measured on each of 15 colonies per plate for each payload. An aggregate of the data from all payloads was pooled and indicated as ‘All’. Black bars = flight; green bars = terrestrial controls. * *p* < 0.001, flight vs. terrestrial.

In addition to quantifying colony numbers, macro-architecture of the colonies was also evaluated. We previously demonstrated that colonies initiated from cells cultured in analog conditions, specifically the ground-based HARV bioreactors, demonstrated a complex and relatively unique morphology referred to as highly irregular wrinkled (HIW) [31]. These colonies also consisted of a high percentage of filamentous cells. We were eager to evaluate whether similar macrostructure was produced from cells cultured in microgravity. As illustrated in Figure 7 (panels A–F), the predominating colony morphology was smooth with agar-invading spike-like projections and larger offshoots, referred to as ‘feet’ projections in the literature [48,49]. This colony structure did not vary between flight samples (Figure 7A,C,E) and corresponding terrestrial controls (Figure 7B,D,F) in the Micro 6 (Figure 7A,B) or Micro 14/FPA (Figure 7C–E) payloads. The observed morphology was not representative of the fully smooth structure commonly associated with terrestrial cultures of *C. albicans* [50] nor to the HIW colonies observed almost exclusively from cells cultured in the HARV (Figure 7H) [31]. The observed spikey morphology seems to be specific to cells cultured in FPAs, as those grown in the FEP bags (Figure 7G) were predominantly smooth, with some colonies harboring the more traditional ‘feet’ projections often attributed to elevated CO_2_ conditions [48,49].

Whether cells produced colonies, and how many, was used to evaluate cell viability as previously described and demonstrated in Figure 6. In addition, the size (diameter in mm) of individual colonies was measured as an indirect indicator of cell health and vigor. The diameter was measured on an average of 15 isolated colonies when possible (there were not that many colonies produced from the non-cryopreserved cells from Micro-14/FEP, Figure 6D). As illustrated in Figure 7I, there was an overall trend for cells cultured in flight to give rise to slightly larger colonies. In conjunction with the yeast growth presented in Figure 3, this is another outcome measure that indicates cells may gain a growth advantage upon exposure to microgravity as compared to terrestrial controls. In addition, the growth advantage appears to be retained for at least a few days following re-introduction to Earth’s gravity environment. Colonies derived from cells harvested from FPAs (Micro-6 and Micro-14/FPA) were similar in size to those harvested from FEP bags (Micro-14/FEP) suggesting the hardware was not introducing differences in growth potential.

### 3.7. Susceptibility to Antifungal Agents

**Figure 8:**
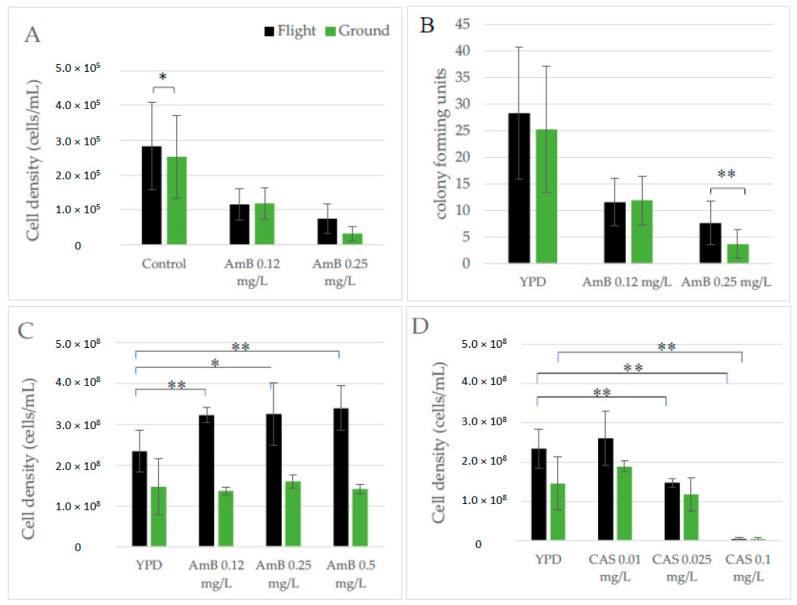
*C. albicans* susceptibility to antifungal agents. Micro-6 and Micro-14/FEP were designed to include analyses of the susceptibility of yeast to antifungal agents during cultivation in microgravity. Both payloads included amphotericin B (AmB) at concentrations previously determined to represent a functional range; the Micro-14/FEP payload also contained caspofungin (CAS) within a functional range. (**A**) Micro-6 samples were activated Early in the mission in the presence of 0.12 mg/L and 0.25 mg/L AmB and control conditions (YPD + DMSO), fixed at the end of the growth period, and cell counts performed upon return, (**B**) Untreated Micro-6 cells that were activated Late in the mission and returned viable were analyzed for antifungal sensitivity (AmB) at the conclusion of the mission using CFU analysis following drug exposure. Micro-14/FEP samples were cultured in the presence of (**C**) AmB at 0.12 mg/L, 0.25 mg/L, and 0.5 mg/L AmB and control conditions (YPD + DMSO) or (**D**) CAS at 0.01 mg/L, 0.025 mg/L and 0.1 mg/L CAS and control conditions, frozen on-orbit, and cell counts were completed following the mission. * *p* < 0.05, ** *p* < 0.006, Flight vs. Terrestrial (**A**,**B**); treated vs. sham (**C**,**D**).

As an opportunistic pathogen, the susceptibility of C. albicans to antifungal agents is an important consideration with regard to spaceflight crew safety. This becomes even more critical based on our previous findings that yeast cultured in analog bioreactors (HARV) have an increased resistance to AmB [31]. As such, the ability of C. albicans to grow in the presence of two antifungal agents (AmB and CAS) was evaluated both by treating cells during the mission while in microgravity, as well as on yeast returned live following the conclusion of the flight. The Micro-6 payload was specifically designed to evaluate yeast growth in the presence of AmB while in microgravity. Cell growth was activated by the addition of medium containing 0.12 mg/L and 0.25 mg/L AmB and the samples were fixed following the growth period. Upon conclusion of the mission cell counts were performed to indicate the susceptibility of C. albicans to the cytotoxic effects of the antifungal agent (Figure 8A). AmB susceptibility was also evaluated in cells returned viable and exposed to drug following the mission, using CFU as the viability indicator. Interestingly, the AmB susceptibility results from the Micro-6 payload were nearly identical, regardless of whether drug exposure was in microgravity (Figure 8A) or following post-flight cultivation (Figure 8B). Although both doses of AmB impacted yeast growth, flight cells were more resilient than terrestrial control cells in the presence of the highest dose of AmB (0.25 mg/L). The Micro-14/FEP payload was also designed to evaluate antifungal sensitivity in microgravity. Parent cultures were serially passaged as described in Methods. On two separate occasions during the serial growth (following day 1 and 3 for AmB or day 2 and 4 for CAS), a dilution of the Large FEP bag culture was used to inoculate Small FEP bags containing antifungal agents (see Figure 1 for experimental design details). On each of the four days of the analysis, yeast were also cultured in control medium and 0.25 mg/L AmB for a direct day-to-day comparison. There was not a notable day-to-day adaptation of yeast to the antifungal agents (data not shown); therefore, samples conducted on the two separate days were treated as replicates and grouped in the analyses described herein. Throughout these studies, there was a consistent trend for the cells cultured without drug to grow to a higher density in flight as compared to terrestrial controls, as demonstrated in Figure 3. In addition, and as illustrated in Figure 8C/D, cells grew to a higher density in all conditions of the FEP payload when compared to Micro-6 FPA samples. Cultures propagated in microgravity were consistently more resistant to AmB than comparable terrestrial controls (*p* < 0.006). As illustrated in Figure 8C, flight samples grew in the presence of AmB when compared to untreated flight samples (*p* < 0.05), whereas the growth of terrestrial samples was stagnated in the presence of the antifungal agent when compared to the untreated ground controls (*p* > 0.33). As such, cells cultured in microgravity were more resistant to all doses of AmB than were the corresponding terrestrial control cells (*p* < 0.002). In contrast, companion cultures in the Micro-14/FEP payload that were exposed to a functionally distinct antifungal agent, CAS, were susceptible to increasing concentrations of drug, (Figure 8D). When CAS-treated samples were compared to the corresponding control cultures (flight CAS vs. flight YPD; terrestrial CAS vs. terrestrial YPD), the lowest dose had very little impact on cell growth and the highest dose prevented nearly all growth. Only at the intermediate dose of CAS (0.025 mg/L) was there an apparent difference between the impact of the drug on cells cultivated in flight or terrestrially. When compared to the untreated samples, terrestrial controls were more resistant to CAS than the cells cultured in microgravity, although not to the level of significance. Consequently, the resistance to AmB does not appear to indicate a broad spectrum resistance to antifungal agents and thus, the resistance to AmB may help elucidate the molecular and cellular adaptations that are occurring in microgravity.

## 4. Discussion

As increasing numbers of people spend longer amounts of time in space, it is imperative to understand the physiological adaptations that humans may experience as well as the threats they may encounter. *C. albicans* is important in its own right as an opportunistic pathogen, but as a eukaryotic organism, it may also provide a simple system that affords insight into how more complex eukaryotes respond to the environment of microgravity. That said, a thorough and robust investigation of biological adaptation to microgravity is complicated for a variety of reasons. Importantly, microgravity cannot be replicated on Earth. Devices that have been developed as simulation bioreactors have not been fully evaluated to determine which aspects of microgravity they simulate, nor the reliability and predictive value they provide. Secondly, opportunities to conduct experiments in true microgravity provide challenges in terms of cost, opportunity, procedures, and outcome measures. Whether utilizing the ISS, suborbital platforms, or free-flyer independent payloads, the conditions, processes, and hardware tend to be unfamiliar and different from those used to establish the foundational studies on which the experimental concepts have been developed. Finally, payload numbers and size tend to be limited, thereby constraining replicate numbers, ability to repeat experiments, and the breadth of experimental variables that can be tested. As such, we feel very fortunate to have flown *C. albicans* on several missions, allowing for comparison of a subset of outcome measures, across flight opportunities, in different types of hardware, and with reference to results obtained in the HARV bioreactor as a simulation device. 

When planning flight experiments there are mission-dependent events that will frame the overall timing of experimental procedures and processes, and importantly, the health and wellness of biological samples. This is a feature rarely encountered in normal ground-based experiments and must be thoroughly considered when planning flight payloads. Based on our experience, the time during which samples must be self-sustaining and without active intervention has shortened, but still involves multiple days. This is evidenced in Table 1 when one considers the time from *launch* to early *sample access*. In SpX CRS-1, this time lapse was 149+ h (6+ days), whereas the shortest lapse was 60 h (2.5 days) during SpX CRS-17. An additional 30–48 h, at minimum, must be added to allow for pre-launch sample preparation, handover procedures, and time required for vehicle packing. Cumulatively and regardless of the experimental design, there is likely to be a multi-day period during which the samples must be stable without crew intervention. At the other end of the mission, sample return timelines may also be critical for some studies. One can expect a multi-day travel period from when the samples leave ISS until they are available for in-lab processing. Beyond the launch and landing time constraints, there is a fair amount of flexibility for sample processing during the mission. Overall mission length of the SpX CRS missions has been fairly consistent and range from 21–36 days, with an average of 28 days. Most experiments are likely to be conducted in a fraction of that time but they all need to be well choreographed, in terms of crew time and equipment availability, with the other research and priority activities being performed on the ISS. The combination of complex timing issues and the use of novel hardware and procedures requires significant planning and modeling beyond that usually employed for standard experiments. 

Although each of our payloads was largely developed independently, we embraced the opportunity to compare basic cellular characteristics of *C. albicans* cultivated in microgravity across multiple missions (see Table 3 for a summary). The characteristics chosen for comparison are primarily those associated with virulence, including growth, cell and colony morphology, and antifungal resistance. In each of the payloads, yeast grew to a higher density when cultivated in microgravity. Surprisingly, the calculated generation time increased as the inoculum level rose, likely due to samples reaching a maximal density sooner, only to reside in stationary phase through the remainder of the growth period. Only in the FEP bags was the planktonic, ground-based, and generally accepted doubling time of 90–120 minutes approximated [51]. These data are limited to end-point analyses since kinetic studies were not performed; therefore, the contributions of rate of growth versus length of time in stationary phase cannot be distinguished. A potential secondary effect of yeast growth is the impact that metabolic activity might have on the surrounding microenvironment, amplified in microgravity due to low fluid shear and lack of convection and bulk flow. Further studies are required to determine whether the differences in growth between flight and terrestrial controls may be explained by a cellular impact on the surrounding microenvironment, including changes in the concentration of nutrients and waste products [34,52]. In addition, *C. albicans* produces significant levels of metabolic gas, such that the pressure within bioreactor units is substantially increased. The impact of such increased pressure on cell behavior has yet to be analyzed. That said, an increased cell density of nearly 10-fold was observed in the FPA hardware of Micro-14/FPA when compared to the Micro-6 FPAs. Whether the gas bubble incorporated into the Micro-14 FPAs impacted access to oxygen or provided a headspace to buffer the buildup of pressure is unclear. It is also worth noting that the mid-mission storage times and temperatures varied among the payloads and may have contributed to viability and ultimately cell density. To inform the impacts of these variations, pre-flight, ground-based viability studies had been previously performed with yeast stored in water. Cells remain sufficiently viable during long term (~30 days) storage at ambient temperature, although viability was highest when stored at 12 °C and next highest at 4 °C (unpublished studies). From these results, it seems unlikely that the modest changes in storage temperature between flight and terrestrial samples contributed to the observed difference in cell density. These results also led to flexibility for the pre-growth storage temperature to simplify flight operations and alleviate the need for on-orbit temperature maintenance equipment, which is often in high demand. 

Unlike many cells, *C. albicans* is fairly recalcitrant to temperature variations. As such, *C. albicans* also retains reasonable viability when frozen in the absence of a cryopreservation agent. Surprisingly, the data presented in Figure 6D demonstrates that the resilience is compromised in cells frozen during spaceflight when compared to similarly treated terrestrial controls. The difference in survival was observed in samples frozen in FEP bags (Micro-14/FEP) as well as FPAs (Micro-14/FPA; ~6-fold fewer colonies in flight than terrestrial controls), demonstrating this is a feature consistent across two types of hardware. We considered the difference in survival secondary to differences in the rate of freezing. Cells are likely to freeze more slowly in the low convection environment of space, but a slower rate is typically considered to be advantageous for cell viability [46]. It was also noted that cells subjected to spaceflight experience more post-undocking temperature transitions than the terrestrial controls. Mimicking these temperature transitions in ground-based studies resulted in a loss of cell viability, but not nearly to the extent of that observed in the studies described in Figure 6D. Membrane integrity is associated with freeze stress; therefore, we are considering adaptations to the microgravity environment that might influence membrane composition. 

In addition to cell growth and survival, we analyzed the multicellular characteristics of *C. albicans*. A complex colony structure (HIW) had been observed in simulation experiments using the HARV bioreactor; therefore, viable cells were plated upon conclusion of the spaceflight missions to determine whether this morphological transition was also triggered in true microgravity. As demonstrated in Figure 7, HIW colonies were not observed following 24 h of growth in microgravity. Interestingly, a minimum of 5 days in the HARV was required to produce the complex colony microarchitecture observed [31], yet those cells went through a daily subculture procedure which may have delayed adaptation to the low fluid shear environment. It did not seem unreasonable to consider that a shorter term exposure to true microgravity may trigger similar adaptation responses as observed in the extended HARV studies. Longer term spaceflight studies are currently underway to address whether this colony morphology transition simply requires longer exposure to the true microgravity environment. Although the analysis of colony morphology required cells to propagate for several days in post-mission gravity conditions, the HARV-induced HIW morphology had been quite stable. Re-adaptation to 1*g* and subsequent loss of complex colony morphology does not seem a likely explanation for the observed lack of complex morphology. Biofilm communities were also undetectable in these studies, again suggesting the timeline of cultivation (24 h) was not sufficient for the development of complex macrostructures. 

Complex colony morphologies of *C. albicans* are typically associated with an increase in filamented cells in the population [50]. Therefore, since complex macro-architectures were not observed in the studies herein, it is not surprising that cell filamentation was also found to be minimal. Interestingly, microarray studies published as part of the microgravity experiment conducted on STS-115 identified the upregulation of several genes associated with filamentous forms of the yeast, including *ALS1*, *ALS4*, *YTH1*, *SPT5* and *STI1*. Other genes associated with filamentation were either downregulated or unchanged, namely *ALS2*, *TEC1* and *ALS2* [35]. Of particular interest, the expression of form-associated genes was consistent with the observed low level of filamentation, specifically the gene coding for hyphal wall protein (*HWP1*) was unchanged in flight samples (−1.09) and yeast wall protein (*YWP1*) was elevated in flight samples (2.1). Current and ongoing studies will contribute to a more comprehensive understanding of differential gene expression as related to *C. albicans* cultivation in microgravity.

One adaptation that has been fairly consistent among flight opportunities and in the HARV simulation experiments is an increased resistance of *C. albcians* to AmB (Figure 8 and [31]). In spaceflight, the level of resistance is greater when cells grow to higher densities (FEP bags), although minimal dose dependence was observed in these same studies. It appears as though aqueous solutions of AmB are less stable than CAS, which may contribute to the difference in dose dependency observed between the Micro-6 (10 days of 4 °C storage following medium preparation) and the Micro-14/FEP (17 days of storage following medium preparation) experiments, although this loss of function was not predicted by pre-flight testing. A preliminary explanation is that AmB lost some efficacy during storage, resulting in drug levels that kept the growth of terrestrial samples in check, but had a milder impact on cells already observed to grow to a higher density in microgravity. The yeast susceptibility to CAS indicates that antifungal resistance is not broad spectrum and the difference between CAS and AmB may provide clues as to the molecular pathways that accompany the adaptation processes to microgravity. Specifically, we are evaluating the role of ergosterol in the adaptation process as it is described as being a cellular target for AmB cell association and pore formation [53].

Cell and colony morphology can be used as indicators as to the composition of the local environment since environmental stresses often lead to morphological transformations, including filamentation and colony extrusions [17]. As observed in Figure 7, cells from this study produced colonies with a spikey (or foot projection)-style colony morphology often associated with cells exposed to environmental stresses, such as increased levels of CO_2_ [8,54]. These studies suggest that cells cultivated in microgravity may produce and then respond to a microenvironment enriched in CO_2_ and other waste products. Since both flight and terrestrial samples demonstrated the colony projections, it will be important to parse out the contributions of microgravity relative to the environment created within specific hardware. Indeed, whether different types of flight hardware modulate or mask the effects of microgravity is an important consideration in these and other spaceflight studies. There exists a limited, but useful inventory of spaceflight-compatible hardware within the industry, with more being developed continuously. Identifying features that are necessary for successful operations is important and potentially challenging. In the case of *C. albicans*, hardware choices were constrained by safety precautions related to potential virulence and by the metabolic gas production during growth. This gas production precluded the use of several hardware models because the integrity of individual compartments was routinely compromised by the internal pressure created by metabolic gas. Thus, hardware that provided significant replicative capacity was restricted (for example multi-well units could not withstand the internal pressures produced by the yeast gas production) and some in-flight analyses were deemed less likely to succeed (microscopy, optical density measurements) due to the presence of extensive bubble formation. Thus, hardware choices for microgravity experiments need to be well-vetted and compromises may need to be made in the name of safety and feasibility. It is in this context that the data herein must be interpreted. With limited numbers of replicates, and the corresponding distribution of values, it was encouraging to identify consistent trends in the data. As summarized in Table 3, cells grew to a higher density in microgravity and produce slightly larger colonies than did the terrestrial controls. There was also a consistent trend toward increased resistance to the antifungal agent, AmB. The yeast did not exhibit a filamentation response regardless of conditions, and the colony architecture was not different between samples cultivated in microgravity and the terrestrial controls. We noticed that when there were differences between flight and terrestrial controls, those differences were amplified when the cells grew to a higher density, especially in the FEP bag hardware. 

The complexity associated with spaceflight experiments and the challenges associated with them dictates the importance of determining whether, and to what extent, ground-based simulators can be predictive of cellular adaptation to the microgravity environment. As such, it is important for investigators to continue exploring biological systems in a variety of spaceflight hardware and to relate those studies to each other as well as ground-based simulators. Comprehensive analyses of these systems in space and on Earth will provide a guide for the predictability of ground-based models and a foundation for investigators to evaluate flight hardware. 

## Figures and Tables

**Table 1:** Mission-specific timeline details for four separate experiments.

	SpX CRS-1	SpX CRS-4	SpX CRS-16	SpX CRS-17
	Micro-6	Micro-8	Micro-14/FPA	Micro-14/FEP
Scrub delay (days)	0	1	1	4
Launch date	8 October 2012	21 September 2014	5 December 2018	4 May 2019
Launch to dock (h)	70.5	79.5	69.5	42
Dock to sample access (h) ^	79	25	30	18
Length of mission (days, launch to splashdown)	21	35	39	31
Experiment completion toundock (h)	48	27	120 *	264 ^#^
Splashdown to home lab (h)	70	68	84.5	66

^ Transition to ISS crew and power resources; * Weather related unberth delay; ^#^ Late experiments not requested.

**Table 2:** Generation time of yeast cells cultured in spaceflight and terrestrial control bioreactors.

Sample	Flight-Generation Time (min)	Ground-Generation Time (min)
FPA-Early Low (Micro-6)	155	159
FPA-Early High (Micro-6)	303	303
FPA-Late Low (Micro-6)	163	163
FPA-Late High (Micro-6)	300	310
FPA-Low (Micro-14/FPA)	113	129
FPA-High (Micro-14/FPA)	127	126
FEP-Large (Micro-14/FEP)	95	99
FEP-Small (Micro-14/FEP)	113	118

**Table 3:** Summary of data obtained across missions and in two types of hardware.

Outcome Measure	FPA (Micro-6)	FPA (Micro-14)	FEP (Micro-14)
Cell Growth	F > T *	Trend F > T	F > T
Cell Density	Low	Intermediate	High
Filamentation	Low, no change F vs. T	Low, no change F vs. T	Very low, no change F vs. T
Colony Morphology	No change	No change	No change
Colony Size	No change	F > T	F > T
AmB Resistance	F > T	F > T	F > T
CAS Resistance	NA	NA	F = T
Biofilm	None	Trend T > F	NA
Freeze Stress Resistance	NA	T > F	T > F

* F = Flight; T = Terrestrial.

## Data Availability

The data presented in this study will be openly available in FigShare 10.6084/m9.figshare.14312498.
